# The Hospital. Nursing Section

**Published:** 1906-12-22

**Authors:** 


### The Hospital.

### Nursing Section.

Contributions for "The Hospital," should be addressed to the Editor, "The Hospital" Nursing Section, 28 & 29 Southampton Street, Strand, London, W.C.

No. 1,058.—Vol. XLI SATURDAY, DECEMBER 22, 1906.

### Notes on News from the Nursing World.

### OUR CLOTHING DISTRIBUTION.

Both in quantity and in quality, we rejoice to state, the garments sent to us for distribution this year excelled all previous occasions. It was noticeable also that men's clothing were a good deal more numerous, and that contributors for the most part seemed to have made a point of providing those bigger and more useful garments that are so sorely needed yet do not allow much scope for the artistic taste. But the babies were by no means overlooked, and there was a nice collection of dainty little hoods and frocks to make some small people smart at Christmas. There was one bundle of Shetland wool vests which was a delight both to see and handle. Another bundle of ample proportions contained some warm jerseys for boys, besides cardigan jackets and shirts for men, all varieties of woollen underclothing for women, and a goodly assortment of woollies. Knitted vests and flannel petticoats fought for supremacy from the point of view of numbers, and boys' shirts were rather more numerous that before, though we should like to have had more. Toys were not forgotten by the kind donors, and a big package of old Christmas cards was divided amongst the children's hospitals. Some of those cosy "hug-me-tights" we were delighted to welcome, and warm shawls are always treasures. Boys' nightshirts were conspicuous by their absence, and stockings for both boys and girls, as well as frocks for girls, were not well represented. The whole of the articles were despatched to their destinations on Tuesday. We have to acknowledge contributions from Miss Brewster, Upwey; Miss Margaret Woodhouse, Birmingham; Sister Agatha, West Ealing; E. M. L.; Miss Annice Orme, Queen's Nurse, Montgomery; Miss Strange, Upper Addison Gardens, W.; Nurse Winifred, West Wimbledon; and Miss M. A. Barnes-Groom, Putney.

### HOSPITALS AND PATIENTS' CLOTHING.

In our columns last week a nurse pleaded for some alteration in the regulations respecting patients' clothing in hospitals. She certainly succeeded in showing that she herself was put to a good deal of trouble and inconvenience because the hospital authorities required her to take away her patients' clothing. There are few rules which are not more or less oppressive to somebody. But this week we publish a criticism from the matron of a leading London hospital on her letter, in which the writer points out that the exercise of a little common sense and a trifling expenditure would have enabled "E. W." to have overcome the difficulty. It can readily be understood that unless there is sufficient room, the worry of taking care of clothing is very great, as articles are likely to get spoilt or lost, in which case the patients would make a great stir about the matter. The question is one for hospital committees, not for matrons, to decide; but we have no doubt that many of the latter consider that it would be an advantage to keep the clothes, if only because friends sometimes bring things for patients to go home in that no matron would wish to have about the place.

### UNTRAINED PERSONS IN CHARGE OF WARDS.

At the last meeting of the Lynn Guardians a statement was made by Captain Hervey, Local Government Board Inspector, which, without any additions, proved the urgent need of an alteration in the nursing arrangements. It was to the effect that when he last visited the infirmary wards in the workhouse he found in charge of them two "absolutely unqualified and untrained nurses." It was true, he added, that the superintendent nurse was there, but she had been on duty all day, and it appeared that she would be there most of the night as well. It was not until the close of a long and acrimonious discussion that the Guardians even decided that two certificated nurses should be substituted for the two untrained persons; and they could not then come to a conclusion on the question of increasing the staff. However, it is something that the scandal of unqualified persons being left in charge of cases requiring, as the Local Government Board Inspector affirms, the constant care and attention of skilled nurses, is to cease at Lynn.

### OVERTAXING THE SUPERINTENDENT NURSE.

The nursing staff in the Bishop's Stortford Poor-law Infirmary has hitherto consisted of a superintendent nurse and three probationers. This means that the superintendent nurse has often to be on duty at night as well as in the day, or that acute cases and cases of serious illness are not properly looked after during the night. The result of overtaxing the strength of the superintendent nurse has just been attested by her breakdown in health. The Guardians last week decided to appoint an assistant-nurse, and rejected the idea of promoting one of the three probationers on account of their non-possession of the requisite qualifications. Both the medical officer and the superintendent affirmed that there is no probationer sufficiently well trained to take the position. Of course they did; merely to call one of them an assistant-nurse would in no way remove her disqualifications.

### AUSTRALIAN NURSES AND OUTDOOR UNIFORM.

A few remarks which we made in our issue of September 1 in defence of outdoor uniform, because the custom of wearing it had been strongly attacked in an American monthly periodical, and vehemently vindicated by several correspondents of one of the Australian nursing organs, has provoked from another Australian organ a long rejoinder which suggests that on this question the writer desires to run with the hare and hunt with the hounds. While it is denied that the correspondents of *Una* are entitled to be regarded as representing Australian nurses generally, and these are said to be as a whole apathetic about the question, the *Australasian Nurses' Journal* agrees with us that "there is no reason why the use of outdoor uniform should be treated as either wanting in taste or in any way opposed to the worthiest traditions of the nursing profession." Having thus endorsed the single contention we put forward, our contemporary proceeds to describe "our American cousins" as reserving "their distinguishing garb to be worn when carrying out their professional duties." It is evident that opinion as to outdoor uniform is divided among Australian nurses.

### AN ALLEGED ABUSE OF DISTRICT NURSING.

A serious charge is made in the *Bishop Auckland Advertiser*, of December 13, against the Committee of the Auckland District Nursing Association, founded by the late Bishop Lightfoot for nursing the sick poor in their own homes. It is alleged by "A Twenty Years Resident" that the Committee require the district nurse to attend the wealthy for the reduced fee of 15s. weekly, and are, in fact, "converting a public philanthropic work, supported by public subscriptions and charity, to the use of the gentry." We know that there has for some time been friction about the district nursing in Bishop Auckland, but it is incredible that this charge can be true, and that public subscriptions, largely made up of contributions by the working classes, are employed to ensure cheap nursing for the well-to-do. But the allegation is so calculated to damage the work itself that we hope the Committee will recognise the importance of authoritatively refuting it.

### THE COUNCIL OF THE JUBILEE INSTITUTE.

At a meeting of the Council of Queen Victoria's Jubilee Institute last week reports from the various committees were received. It was stated that during the last quarter Queen Alexandra has approved the enrolment of 53 Queen's nurses. The Countess of Dudley, who was present at the meeting, reported that there is a pressing need for money to extend the work of her Fund for the establishment of district nurses in the poorest parts of Ireland, fourteen districts having already been opened. As we have already mentioned, the Viceregal Commission on Poor-law Reform in Ireland referred, in their recent report, to the remarkable and unquestionable success of this scheme.

### NURSES' BAZAAR AT PLAISTOW.

In consequence of Princess Henry of Battenberg suffering from influenza, she was unable to fulfil her promise to attend the Nurses' Bazaar in aid of the Maternity Charity and District Nurses' Home at Plaistow, which was held on Thursday and Friday last week. The Duchess of Somerset very kindly undertook to perform the opening ceremony in her place, and the Duchess of Rutland was also present. The Bishop of Colchester, who was one of the speakers, referred with great regret to the absence of Sister Katherine, a blank that was felt by all. It had been hoped that she, who was the foundress of the work and the centre of all the activity and enthusiasm connected with it, would be there, but unfortunately it was necessary for her to leave a week or two before. A kindly welcome was accorded by the Bishop to her successor, Miss Macqueen, and all good wishes were tendered to the new superintendent. On Friday the Princess Alexis Dolgoroukie opened the bazaar. The hall was very cold and quite in keeping with the "Ice Carnival" advertised. The nurses bought gaily from each other's stalls when outside purchasers were scarce. Some little cripple children, patients on the District, with spinal or hip disease, were allowed in for a treat to brighten up their lives a bit; and to each the Princess made a little gift and spoke some kind words—a never-to-be-forgotten episode, which will be mentioned with pride and delight by the other children, or sad, hard-working mothers, for many a long day to come.

### PRIZE DAY AT BRISTOL GENERAL HOSPITAL.

On Monday afternoon last week a very pleasant gathering was held at Bristol General Hospital, when Mr. Joseph Storrs Fry, the Chairman, distributed the medals, certificates of merit, and prizes to the successful candidates. Many friends were present, including the matrons from most of the institutions in Bristol, and Miss Jones, matron of the Birmingham General Hospital. Nurse Crease won the gold medal, and Nurses Walmesley and Armstrong the silver medals; certificates of merit were awarded to Nurses Harvey and Rena Smith, and the Lottie Culverwell Memorial prize to Nurse Crease. In physiology Nurse Grahame was first of the second year probationers, and Nurse Harvey second. Nurse Badcock was first of the first year probationers, and Nurses Woolcott and Atkinson tied for the second place. In anatomy Nurse Grahame was first of the second year probationers, and Nurses Webb and Pontifex tied for the second place; Nurse Woolcott was first of the first year probationers, and Nurse Atkinson second. In surgical nursing Nurse Harvey was first of the second year probationers, and Nurse Percy second; Nurse Woolcott was first of the first year probationers, and Nurse Wyatt second. In medical nursing Nurse Pontifex was first of the second year probationers; Nurse Atkinson was first of the first year probationers, and Nurse Page second. The matron's prize was given to Nurse Perry.

### DEVON AHD EXETER HOSPITAL.

The President of the Devon and Exeter Hospital has received from Dr. Steward, of Harley Street, his report of the examination which the latter has recently conducted of the probationers at that institution. In enclosing particulars of the results, Dr. Steward says that it gives him great pleasure to state that he considers that the nurses he has examined have been extremely well trained in all parts of their work, and that the standard of proficiency is a very high one, as is proved by the fact that the last in the list obtained over 78 per cent. of the marks possible. The first in the list was Nurse Dufty, who secured 1,882 marks. Nurse Langford was next with 1,843, and then followed Nurse Fisher, 1,804; Nurse Egremont, 1,797; Nurse Barrable, 1,786; Nurse Pyle, 1,776; Nurse Jackson, 1,741; Nurses Gardiner and Dash, 1,738; Nurse Barker, 1,733; Nurse Pardoe, 1,726; Nurse Mallett, 1,669; and Nurse Cooke, 1,646.

### THE MILITARY NURSING SERVICE IN INDIA.

We are officially informed by the Military Secretary of the India Office that Miss M. E. Tippetts, Miss K. Cawley, and Miss K. O'Brien have been appointed nursing sisters in Queen Alexandra's Military Nursing Service for India. Miss Tippetts was trained at Guy's Hospital, Miss Cawley at the Poplar Hospital, and Miss O'Brien at the South Infirmary, Cork.

### ROYAL NATIONAL PENSION FUND FOR NURSES.

The offices of the Royal National Pension Fund will be closed on Monday, December 24, as well as on Christmas Day, December 25, and on Boxing Day, December 26.

### SALE OF WORK AT HARROGATE.

The handsome sum of £93 was realised by a sale of work last week at the Clovelly Nursing Home, Harrogate. The sale was entirely got up by nurses with the view of assisting to found a cot in Harrogate Infirmary, for which a sum of £500 is required. The Matron presided at the "Forget-me-not" stall, and nurses at most of the others. As it was not expected to raise more than £50, the result must be particularly gratifying to the workers.

### CERTIFICATES AND COOKERY.

At the examination held under the auspices of the Royal Victorian Trained Nurses' Association on October 24 the following matrons were successful: Miss Allan, Williamstown Hospital; Miss Anderson, ex-matron Perth Hospital; Miss Benallack, Maryborough Hospital; Miss Campbell, Mooroopna; Miss Conyers, Queen's Memorial Infectious Diseases Hospital; Miss Corrigan, Colac Hospital; Miss Davidson, Geelong Hospital; Miss Dewar, ex-matron Queen Victoria Hospital; Miss Hill, Milton House; Miss Hooley, Hamilton Hospital; Miss Lewers, ex-matron Warrnambool Hospital; Miss Mann, Women's Hospital; Miss Mitchell, St. Arnaud Hospital; Miss Robertson, Stawell Hospital; Miss Reynolds, Gippsland Hospital; Miss Samsing, Queen Victoria Hospital. Before claiming their certificates, Misses Campbell, Conyers, Hill, and Lewers will be required to qualify in cookery.

### HOSPITAL NURSES AND ADVENT SERVICES.

At one of the special Advent services in the chapel of St. Mary's Hospital, on Thursday last week, Nurse Thorpe sang Wilson Barrett's "Shepherd of Souls" from "The Sign of the Cross," and Nurse Dawson "The Gleaner's Slumber Song." The latter was followed by the Welsh setting of "Lead Kindly Light," sung as a duet by Nurses Hall and Sherwood. The night nurses, and the day nurses who were off duty, mustered in force at the service.

### THE POPULARITY OF COTTAGE HOSPITALS.

We understand that no fewer than 134 applications were received for the post of Matron of Fleetwood Cottage Hospital, to which, as we announced last week, Miss Godson has been appointed. Her qualifications are excellent, but we may fairly assume that many of the other candidates were not appreciably less eligible. Fleetwood is a pleasant and rising town of Lancashire, on the coast. The hospital contains fifteen beds, and the nursing staff consists of the matron and two nurses. The number of applicants for the matronship shows the increasing popularity of cottage hospitals among nurses.

### THE SOCIAL UNION AT BRISTOL INFIRMARY.

The fourth autumn meeting of the Nurses' Social Union was held recently in Bristol, and by the kindness of the Committee and Matron the members in that centre of the Union were invited to the Royal Infirmary. The medical and nursing staff gave their guests a courteous welcome, and succeeded in making the occasion agreeable as well as instructive. The wards, kitchen, bacteriological laboratories, and anatomical museum were inspected, and a short address was given by Mr. Munro Smith, a member of the visiting staff. His words showed so much sympathy with the importance of a nurse's task, and such knowledge of the difficulties that beset her, that they could not fail to have been helpful. He also expressed the hope that the Nurses' Social Union has a great future before it. Tea was served at little tables in the dining-room of the Nurses' Home. The guests then adjourned to the sitting-room, and finished a pleasant day most pleasantly by listening to a short but well rendered programme of songs and pianoforte music by members of the nursing staff.

### THE CONVICTION OF "NURSE" MILLER.

The woman, Miller, who last week was tried at Leeds Assizes on eight charges of illegal practices, found guilty of felony, and sentenced to five years' penal servitude, was described by counsel for the prosecution as having "formerly been a professional nurse." But not a word was said by him either respecting her training or her professional experience. The probability is that as she conducted what she called a private maternity home and called herself "Nurse" Miller, her claim to belong to an honourable profession was not subjected to any inquiry, but was taken for granted.

### The Nursing Outlook.

"From magnanimity, all fears above;
From nobler recompense, above applause,
Which owes to man's short outlook all its charm."

### THE HUMANE INFLUENCE OF THE MODERN HOSPITAL.

It has been said with some force and truth that the present age, in this country, is too emotional, and that the so-called up-to-date newspaper demonstrates the fact every morning. No doubt, if money-making were the whole aim of man's existence, emotion would be out of place, for those who had none of it would speedily extract all the money from the pockets of their fellows. In human affairs, especially at Christmas time in a Christian country, if people are to live and not merely to exist, they must cultivate their higher nature habitually. The higher nature demands sympathy, which is nowadays held by many to be a weakening force. But sympathy is the leaven of social life, and those who possess it in the truest sense are deservedly amongst the most popular and happy of mankind. It is undoubtedly true that England to-day needs waking up. The average man and woman would be all the better for more discipline and less introspection; more generous sympathy and less selfish narrowness; more striving after ideals and less contentment with the commonplaces in everyday existence. Realising all this we rejoice that Christmas is once more with us, and we wish all our readers a happy and joyous Yuletide of the old-fashioned kind. They, for the most part, have to do with palaces of pain, the sick-room, and the solace of the miserable and suffering. Yet we make bold to say that Christmas will probably mean more to them and bring to them more real joy in their actual work, and as individuals, than to almost any other class of the population. Why the modern hospital is such a splendid national asset in this country, is because of its humane influence for good.

The humane influence of the modern hospital is not believed in by any, probably, except those who are familiar with its work and life. It has, at any rate, succeeded in extending its influence to all classes of the community, and there never was a time, in the history of this country, when so many people were genuinely and personally interested in the work of the hospitals. The interest to which we refer is wholly divorced from the medical and surgical aspects of the hospital, and its relation, in these respects, to each individual member of the population. Their Majesties King Edward VII. and Queen Alexandra, with all the members of the royal family from the Prince and Princess of Wales downwards, give every year from conviction and as a matter of duty, and also, we believe, as an expression of genuine thankfulness for the blessings of health, which they enjoy, a great deal of time, mind and thought to hospital work and needs. The League of Mercy has been founded upon the principle of personal service in the days of health in the cause of the sick. It has enlisted an army to be now counted by hundreds of thousands of people, who deliberately and earnestly devote a portion of their time, each year, to work for the sick. These facts illustrate, that if, in one sense, sympathy is a weakening force, in another and higher sense, it moves individuals to exercise self-denial, and to recognise their dependence upon a higher power. Hence this Christmastide should be a time of great rejoicing and stimulus to hospital workers and to members of the medical profession, for the outside world, to an increasing extent each year, is in direct sympathy with the sick and suffering and their attendants, who find a home in our modern hospitals.

Each well-administered modern hospital is a little republic in itself. Every worker who is worth anything becomes absorbed in the duties and devoted to the endeavour to make his or her portion of the work efficient. Each has its own social centres, where good-fellowship and kindly intercourse can be promoted and enjoyed with a minimum of wear and tear. We have lived in a big hospital for probably more years of a busy life than almost anyone else, and we know, that, there is nothing more inspiriting and enjoyable than the social life of a great institution, where the responsible chiefs are animated by sentiments of justice and where the spirit of the place is to secure the maximum of efficiency in the work, and the maximum of comfort for the suffering. Hence the humane influence which the hospitals exercise upon everybody from the physician to the patient.

Christmas Day in a modern hospital is an experience which no intelligent man or woman in this country should miss. To the staff it is probably the hardest, as it is often the happiest day in the year. Each one's desire is to make the patients thoroughly happy and forgetful of their sufferings. And then, too, how joyous a thing for friend to meet friend, and in friendly competition to strive to make each ward or section the brightest and happiest of all. Such devotion deserves grateful recognition from the whole nation, and we hope each may receive it in a practical form, everywhere, this year. The workers themselves, we are certain, will greet each other merrily and have a joyous time, animated, however unconsciously, by the feeling, that the day has been well spent, and that this Christmas will always remain a red-letter day in their lives. We wish each one of them the brightest of Christmas seasons and a prosperous New Year.

### The Care and Nursing of the Insane.

By Percy J. Baily, M.B., C.M.Edin., Medical Superintendent of Hanwell Asylum.

### II.—NURSING THE SICK.

(*Continued from page* 149.)

### *Moving Helpless Patients.*

As in all other matters there is a right and a wrong way to move a patient who is too weak and exhausted to help himself. A nurse should never attempt to lift a patient who is obviously too heavy for her, without assistance, and in no case should the arms be made use of as handles as it were to the patient. If an attempt be made to lift a helpless patient by the arms there is great risk of dislocating the shoulder or fracturing the humerus.

In most cases of severe illness there is a constant tendency for the patient to slip down in the bed, and the more exhausted and helpless he is the more marked is this tendency. When this happens the patient becomes most uncomfortable and he must be lifted by the nurse back on to the pillow. If the patient be not too heavy and is able to render the nurse a little help she may be able to lift him back without other assistance. He should put his arms round her neck while she passes one hand well beneath his shoulders and the other beneath the sacrum when the lifting will be fairly easily done. If the patient is too ill to render any help and too heavy for one to lift, the nurse should be on one side of the patient and her assistant on the other, and, as before, one hand is to be passed under the shoulders and the other beneath the sacrum while the assistant places one hand beneath the back and the other under the patient's knees. When a patient has to be lifted from a stretcher or operating table on to a bed the head of the stretcher or table must be placed at the foot of and in a line with the bed, so that when he is lifted by two (or more) nurses, as described above, they can easily pass him along by taking a few side steps and lower him gently on to the bed.

### 5. Bed-sores.

It should be the constant effort of the nurse who is in charge of a sick ward to prevent the occurrence of these terrible and unsightly wounds. It is true that they will occasionally occur in spite of the greatest possible care, but on the other hand there is no other misfortune in the whole range of nursing which is so certain to be met with as this where there is the slightest continual neglect or carelessness, and when they occur constantly in a ward it is fairly conclusive evidence that the nursing is bad.

"A bed-sore is an inflammatory condition of the skin and underlying structures which results in the local death of the tissues affected." They are naturally much more difficult to prevent when the patient is insane than in sane people. They are liable to occur in all cases where the vitality of the patient is greatly reduced by senility or any chronic disease which entirely confines the patient to bed, especially if he be suffering from disease of the nervous system or is greatly emaciated. They are also met with in some cases of acute exhausting disease like typhoid fever, but probably in such cases invariably indicate bad nursing.

The causes of bed-sores are either (1) general, or (2) local.

The general causes are all those conditions which lower the vitality of the patient and include all exhausting diseases, such as phthisis, cancer, chronic heart and kidney disease, diseases of the nervous system, and senility.

The local causes are as a rule more or less preventable. Among them may be mentioned pressure, irritation of the skin due to urine or fæces or the retention of sweat, as where two portions of skin come into contact with one another, or the presence of crumbs in the bed or rucks in the under sheet or draw sheet, bruises or excoriations of the skin such as may be produced by the careless use of bed-pans, etc.

Bed-sores occur generally over the bony prominences, such as the sacrum, hips, knees, ankles, and spine, and are often found at the spot where the limbs come into contact with one another if the patient is allowed to lie with one leg crossing the other.

There are two varieties of bed-sore which are (1) The acute bed-sore, (2) the chronic bed-sore.

The acute bed-sore is not common. It occurs only when the brain or spinal cord are injured or diseased. In asylums for the insane it is found chiefly in cases of general paralysis, especially after convulsive atacks. It is probably due, at least to a large degree, to an interference with the nutritive function, which we have already seen is exercised by the nervous system over the tissues. Some slight injury to the skin (abrasion or scratch) which may be received by the patient during the convulsions may possibly be the starting point of the bed-sore. The sore generally commences by the formation of blisters on the skin which rapidly becomes red and inflamed and dies. The dead portion forming what is called a slough then gradually ulcerates away leaving a more or less extensive hollow wound.

The chronic bed-sore forms more slowly than the acute variety. It is this form of bed-sore which careful nursing largely if not entirely prevents and such wounds are fortunately becoming less and less common as the training of asylum attendants advances. It commences by the skin of the part becoming a dusky red or purplish colour from which the cuticle soon gets rubbed off. The affected portion of skin dies forming a tough leathery blackish or greyish black slough which finally separates and comes away leaving, as in the acute bed-sores, a deep and extensive wound which may expose the muscles and even the bones and ligaments.

In both forms of bed-sore the slough will almost certainly become septic (putrid) and have a very foul smell. If this should happen the products of the putrefactive change may be absorbed by the blood producing a form of blood poisoning which results in a high temperature and often death. Bed-sores may also occasion death by the drain upon an already exhausted system which is occasioned by the frequently profuse discharges which come from them.

### The Nurses' Clinic.

### THE DISTRICT NURSE AND THE PREVENTION OF DEFORMITY.

In trying to prevent the creation or aggravation of disabling deformities among her patients and their families, the district nurse has more to struggle against than mere poverty. There is the moribund, but occasionally active, prejudice that children are "born so," and that it is useless, if not impious, to try and change immutable fate. There is also the conviction of slightly more advanced parents that the one and only cure for every kind of deformity is a rigid support. Thirdly there is lack of intelligence in applying the means suggested or provided by the surgeon. Lastly, and chiefly, there is utter ignorance of the contributing causes of deformity.

If the district nurse's work is to be on useful lines, she must first consider the origin of deformity, secondly the special development of the malformation, whether spinal curvature, hip disease, etc., and thirdly the best means of combating the deformity, actual or probable. In some cases the first essential is change of diet, in others rest, in others regulated exercise and abundance of fresh air.

An enormous proportion of infants even in the poorest and most insanitary homes are born with well nourished bodies, and no obvious personal defects, but mistaken treatment and bad housing in many cases rapidly produce weaknesses that may end in malformation more or less seriously affecting the whole life, both as a citizen and a private individual. The very way in which the child is held may affect every organ of its body. The human framework is a most complicated whole of which the interdependence of even the parts apparently disconnected can be clearly proved, and such a thing as a strictly limited and isolated loss or injury cannot exist. Anything that weakens the structure at any point, or that prevents it from expanding when the time for expansion has come, acts and reacts upon the whole body, and is a direct cause of deformity. Insufficient or innutritious food injures the whole structure, bony, muscular and nervous; scanty or unsuitable clothing throws undue strain on the organs; bad air lowers the vitality and disinclines the child for the active exercise essential to his well-being.

It is exceedingly difficult to make mothers believe that simple ignorance of hygiene on their part causes most of the deformities in their children. They tell you that Tommy has hip disease because a naughty boy knocked him over a form at school, and that Harry died of meningitis because a snowball struck him on the head. They do not seem able to realise that these trifling accidents were occasions, not causes, and that if Tommy and Harry had been moderately well-fed and well-cared-for little boys, they would first have howled and then tried to make their assailants howl, their respective sisters would have threatened to tell their respective mothers, the pupil-teacher would have slapped one or both, and that would have been an end of the matter.

Again, among the poor small injuries are often neglected and lead to worse ones, and these to worse still. A child has a bad chilblain, the hard boot rubs on the sore, the sore is poisoned, the poisoned wound is left undressed, and amputation or permanent stiffening of the leg results. I have known an arm lost up to the shoulder joint owing to an unchecked habit of nail-biting. Or a child "ricks" its ankle, and instead of being compelled to rest for a few days, it is allowed to hobble about. To avoid the pain of using the ankle joint it adopts a method of walking which first affects the knee injuriously, then the hip, then the spine, and perhaps ultimately the brain.

Another thing that the nurse has to be on the constant look-out for is a too prolonged use of treatment that may once have been desirable, but which is continued until it becomes a source of injury more serious than the original complaint. A young child, for example, has been dismissed from hospital with some part of its body encased in plaster of Paris, and with strict instructions to return to the doctor in so many days or weeks for examination. If no one intervenes, the days may be weeks, the weeks months, and the joint stiffens. There are in addition innumerable cases where a child has been provided with a mechanical support by the Orthopædic Hospital, or some other charitable association, and for lack of intelligent supervision is allowed to wear the instrument long after he has outgrown it, or when the adjustment plainly needs altering, or after the most essential part of the contrivance is lost or worn out, or when it is in a condition to cause chafing and blisters. To mention the most simple, everyday appliance : can anyone walk through the poorer quarters of a large town without observing children who are using crutches as much as six or eight inches too short for them, and unsupplied with proper grasping bars? Again, there are children who have the right supports and in good order, but whose parents are too uninstructed to apply them properly, the children cry, and complain, and the most expensive gifts of a charitable public are thrown on one side as "doing more harm than good to the poor little dears," and incurable deformities result.

Mothers need special instruction as to the class of deformities caused by weakening of the muscles, and the extreme value of simple, regulated exercises must be insisted on. It will be uphill work—but it is that in every class of life. "Leaving it to nature" is a plan that appeals to the laziness of mankind, while directly thwarting nature may be accepted as a duty, and is to some minds almost a pleasure; but to learn to understand natural processes and try to assist them is to most of us a heavy and unwelcome task! A little very elementary teaching of anatomy may sometimes be the shortest way home : how is a mother to understand the importance of strengthening the spinal muscles when she probably believes—many far more expensively educated people also believe—that the spine is all in one piece, and the more rigidly immovable it is, the better? Or how can she grasp the need for expanding the chest if she knows nothing of the nature of the lungs? A lad, educated at one of the oldest public schools in England, and by no means unintelligent, told me in all seriousness that he had "found out" where his lungs were. I asked him to indicate the spot, and he showed me the bluish under side of his tongue.

If the nurse's work is to be prevention, not alleviation, she must begin at the very beginning and teach mothers that the main causes of deformity are neglect of small injuries and the passive acceptance of insanitary conditions of life, whether arising from air, water, food, clothing, or general habits.

### Presentations.

Rollesby Union Infirmary.—On leaving the post of nurse at the Rollesby Union Infirmary to take up her duties as night nurse at the Hendon Union, Mrs. E. Lewis was presented with a dressing-case and a box of handkerchiefs by the Chairman, the Rev. R. C. Tacon, from the Guardians and officers of the Rollesby Union Workhouse.

### Incidents in a Nurse's Life.

### CHRISTMAS MORNING IN A FEVER HOSPITAL.

As the clear voices rang out

"Hark, the herald angels sing,
Glory to the new-born King"

on the still air, I hurried to the door of my block. There stood a group of day nurses, who had come to give us a treat. It was Christmas Eve, and sister and I were filling the stockings in the large kitchen, for each of our twenty-four small patients had one of these articles ready for Santa Claus. On entering the girls' ward earlier in the evening I had heard a sleepy voice murmur: "Has Daddy Christmas been yet?" but on being assured that this great personage never came while anyone was awake the owner soon slumbered again.

As the carols commenced one small boy, evidently under the impression that there was a fire or an earthquake, sat up in bed and roared.

He was, however, speedily soothed, and several of the children were soon awake and enjoying the singing. After requesting numerous impossible articles and being offered "a penny to go into the next street" the carol singers made their way to the next block, and sister went round with her wonderful basket.

At last she departed with a cheery "Good night," and I was left to await the dawn of Christmas Day. As I gazed around I was reminded of a fairy scene, although the gentle snores I heard were very human. Pink paper and trailing ivy decorated the brackets and tables, and across the wards hung garlands of evergreen, from which were suspended Chinese lanterns and artificial birds. The doors were draped in crêpe paper fastened with butterflies, and weird brown owls peered out from various corners. The great Christmas-tree stood at the top of the girls' ward, and was a delightful thing to behold, carrying me back in memory to my childhood's days.

Presently came midnight, and as I threw up the window I almost expected to see the Angel Choir and to hear their "Peace on earth, goodwill to men," for the air seemed full of a holy stillness and awe.

Then the night sister came round to wish us all a happy Christmas, and after this the time passed rapidly until four o'clock, when the first small girl discovered that Daddy Christmas had indeed visited her. The wards were quickly alive with happy faces and cries of joy. The musical instruments which were found in some of the stockings added to the merriment and noise. My morning's work was brightened by the sight of the joy around me, and soon eight o'clock arrived and with it the day nurses, who gave us a grand ovation and more carols.

On going off duty the night nurses had a hurried meal and some of us went to church. On returning, we went quickly away to our beds, for we were to rise at 6 o'clock for our Christmas dinner.

The postman arrived late in the day, and a splendid budget was brought up to me, for liberties in the way of sleep were allowed on this day.

With six o'clock up came the day nurses to dress for dinner and to awaken us with gay banter and chatter. Before long we were all assembled in the dining-room and seated around the prettily decorated table, with matron at the head, and dinner proceeded with a great deal of fun and laughter. After turkey and sausages, matron gave us tea in her room, and then we adjourned to the wards to enjoy some games with the children. They were eager to tell the night nurses of the grand dinner they had partaken of, when matron carved and the sisters waited on them, and all the nurses wore the paper caps found in the crackers. I was told that one small boy had remarked to his neighbour, "I have finished my pudding before you." Whereupon the other made answer, "Well, your mouth's bigger than mine." The little ones had enjoyed a very happy day, but were eager for more fun and games. The piano had been taken down to the largest block, and we danced, sang carols, and played "Musical Chairs" until the children's bed-time, when the night nurses collected for a rest and a chat before going on duty once more.

### How to Start a Lending Library for Nurses.

Solomon tells us that "of making many books there is no end"; but there is often an end of reading them for nurses when they leave their training schools, with their libraries, reading-rooms, and other advantages, and take up work in the country, far away, perhaps, from any centre of light and learning. And yet then, if ever, a nurse should read if she wants at all to keep abreast of the times. But the ever-present difficulty of expense comes in. Medical books are notably costly, and if a nurse does save up her pennies to buy one or two, in a few years' time probably fresh discoveries are made or treatments alter and the book becomes out of date. A nurse needs to be within touch of a really up-to-date library, which will contain books on all branches of her profession; but the subscription to it ought to be made within the compass of the slenderest purse.

An attempt has been made in Somerset, which, by its Social Union and other means, does so much for its nurses and their work, to supply this want. A Lending Library containing over 130 books has been formed, and they are added to from time to time; any book that a nurse particularly wishes for being chosen when possible. The library contains all the standard nursing text-books, works on midwifery, gynæcological nursing, anatomy, physiology, children's diseases, massage, first aid, foods and feeding, sick cookery, etc. A nurse may keep a book for five weeks, and if she wishes it can renew it for the whole or a portion of that time. She also sees some of the nursing papers each week. The subscription for both books and papers is 1½d. a week, or 12s. 6d. for the year. As the majority of the nurses in Somerset live in country places, hardly any are able to change their books personally, but each nurse is supplied with a catalogue and writes for what she wants to the Librarian, and at the end of each half-year the sum she has spent in postage is returned to her, so that the 1½d. per week is an inclusive charge. A paper is allowed to every three or four nurses and may be kept three days, and if a nurse has—as sometimes happens—been unable to read it during that time, arrangements are always made for it to be returned to her again if desired. So far an ex-nurse has always been found who has been willing to house the books and act as Librarian, while the papers and general management has been vested in the secretary.

The two greatest difficulties are funds and getting the nurses to forward the papers regularly. At first the latter difficulty occasioned considerable trouble, not only to the nurses who did not get the papers, but to the unfortunate secretary, who was bombarded with complaints, and who had often to write round to four or five nurses before discovering where the stoppage was. But now that the working of the scheme is better understood, and the number of nurses to each paper reduced, delays seldom occur, and one soon learns to know which nurses are quick readers and like seeing a paper first and which prefer it last.

Expense is another problem. Of course, the library does not pay on these terms, but if a small fund is collected to start with, it does not take a great deal to keep it up; but the supply of new and expensive books has often to be curtailed more than one likes. The nurses themselves are very generous. A small sale of work was held last year to which nearly all the members contributed, while many often do not charge their postage and others, who can, give some little donation to the funds.

Friends, too, sometimes help by gifts of books, and any contributions in the way of nursing or medical works would always be most welcome. If more libraries such as these were started one would think that doctors and others must often have old editions of various books for which they have no further use, and which would still serve a useful end if donated to such an institution.

### Hammersmith and Fulham District Nursing Association.

In connection with the Hammersmith and Fulham District Nursing Association, which is affiliated to the Jubilee Institute, Miss Curtis, the superintendent, and her seven nurses gave an "at home" at the Hammersmith Town Hall on Monday last. Quite a large number of people—subscribers, friends, and well-wishers—responded to the invitations sent out, and enjoyed the entertainment provided by the hostess. A varied programme of music and recitations was given in one of the large halls, while in an adjoining room tea, coffee, and refreshments were dispensed by the nurses. During the "interval," which at most concerts is usually a time of boredom, the business of the day was conducted. The Mayor of Hammersmith took the chair, and in a few introductory remarks spoke sympathetically of the work of the Association, which is founded on broad principles, and alluded to the remarkable success of Mrs. Levy as a collector.

The Rev. E. S. Hillard, High Master of St. Paul's School, then gave an address, in which he spoke feelingly of the difficulty which beset subscribers in choosing among the multiplicity of works of mercy for the bestowal of his limited means, and on the old principle of "Charity begins at home," he recommended his audience to support local charities. He was glad to see from the report in his hand, which included a column of "Payments of Patients," that this Association assisted not only the very poor, whose patent poverty carried a more direct appeal to the rich, but also those hard-working men and women who kept up a daily struggle for a decent living, and were glad to help forward, according to their ability, the work which had helped them.

The collecting books and boxes of the year were received by Mrs. Hillard, and fresh ones were given out. A vote of thanks to Mr. and Mrs. Hillard and the Mayor having been warmly accorded, the programme of music was proceeded with. Among the company present were several medical men, whose presence testified to the local appreciation of the work of the Association, which has just completed its seventeenth year.

### The Work of the "Brave Poor Things."

Nothing prettier, quainter, or sweeter, though in a way nothing sadder, could be imagined than the display of work done by the "Guild of the Brave Poor Things," which was opened at 8 Chesterfield Gardens on December 11th by Adeline, Duchess of Bedford. Her Grace appealed for the sum of £1,400 to carry on the work of the Guild, and the Bishop of London, who is the President of it, claimed the privilege of giving the first cheque for the purpose. The origin of the Guild, or at least of its strange name, is to be found in Mrs. Ewing's pathetic "Story of a Short Life," in which the child hero, when crippled, resolves that if he is to be a "poor thing," he will at least be "a brave poor thing," and truly make his own the motto of his family, "Laetus sorte mea"—"happy in my lot." The "poor things" whose work was shown are crippled children, drawn from dreary, and sometimes wretched, homes, and trained at a Home in Chailey to be not merely useful and resigned, but happy. They have adopted as their own the motto, "Laetus sorte mea," and it was to be found painted, carved, or embroidered on all the work shown—on spades and buckets for the use of healthy paddlers at the seashore, on curtains and cushions, on toy wheelbarrows and dolls' cradles. Where the motto was not, the monogram "G.B.P.T." met the eye. The special work of these afflicted children is the provision of articles of use and pleasure for other children. There was admirable nursery furniture, solid in quality and artistic in design—chairs and tables and settles and cupboards—all of a height to be easily reached by a child. There were toys, all as substantial as the furniture—that is to say, likely to stand a far longer period of childish use, not to say abuse, than the average toy of commerce. There were curtains and cot-covers, children's frocks and coats, and all manner of dainty garments for babies, as fine and dainty as any child or its fastidious mother could desire. And all were priced at figures which certainly did not suggest sweated labour, but which were by no means too high for the quality of material and workmanship. And finally, there was the glory of the show, a truly marvellous doll's house, made to the order of Mrs. Emil Mond for her daughter, and forming a perfect model of a Sussex country house. This most desirable mansion opened not merely in front, after the manner of dolls' houses, but at the sides, and displayed an oak-panelled hall with a practicable staircase—practicable for dolls, that is—and besides the ordinary apartments common to all houses, a billiard-room with table well supplied with cues and balls, a schoolroom with desk and globe, and a bathroom with real water laid on—altogether a toy so charming, so complete, and so perfectly finished, that one could not but feel that the making of it had been as great a pleasure to the "brave poor things" as the possession of it could be to the fortunate little maid who is to possess it. The whole display showed how much can be done for the one-armed, the lame, and the feeble by care, training, and affection, and how by bringing the element of joy into the work, that work itself can be improved and the lives of the workers made happier.

### To Nurses.

We invite contributions from any of our readers, and shall be glad to pay for "Notes on News from the Nursing World," "Incidents in a Nurse's Life," or for articles describing nursing experiences at home or abroad dealing with any nursing question from an original point of view, according to length. The minimum payment is 5s. Contributions on topical subjects are specially welcome. Notices of appointments, letters, entertainments, presentations, and deaths are not paid for, but we are always glad to receive them. All rejected manuscripts are returned in due course, and all payments for manuscripts used are made as early as possible after the beginning of each quarter.

### Death in our Ranks.

We regret to record the death, which took place on December 9 quite suddenly, at Newton Abbot, of Miss Charlotte M. Bann, until recently lady superintendent of the Liverpool Royal Infirmary and its Home and Training School for Nurses. Miss Bann was trained at the Manchester Royal Infirmary, and at the early age of twenty-eight obtained the matronship of the Swansea Hospital, which post she filled for three years. She was then appointed to the Birmingham General Hospital, but preferred to accept the post of matron at the Bristol General Hospital, which was offered her almost simultaneously. From that institution she went to Liverpool in 1893, carrying with her testimony as to her exceptional ability and the esteem in which she was held. In 1900, to the great regret of those with whom she had been associated, she was obliged by ill-health to relinquish the work she loved and in which she had shown herself so exceptionally capable. Both at the Bristol General Hospital and at the Liverpool Royal Infirmary she reorganised the system of nursing and greatly reduced the household expenditure.

Miss Bann's amiable disposition, unfailing tact, and intimate knowledge of hospital matters won her the affection and respect of all with whom she was brought into contact. The knowledge that her retirement did not secure her restoration to health was keenly felt by her former colleagues, in whose work she continued to interest herself to the last. We have known few matrons better qualified to fill the higher positions of the nursing world. Miss Bann, like Mrs. Wardroper and other really able women administrators, won her way by quietness of manner, firmness of will, justice of principle, and a genuine enthusiasm in the maintenance of the highest efficiency. She aimed to secure the progressive development of that work entrusted to her control. Miss Bann will be deservedly honoured and missed, but her work will endure.

### Everybody's Opinion.

[Correspondence on all subjects is invited, but we cannot in any way be responsible for the opinions expressed by our correspondents. No communication can be entertained if the name and address of the correspondent are not given as a guarantee of good faith, but not necessarily for publication. All correspondents should write on one side of the paper only.]

### PATIENTS' CLOTHING AT HOSPITALS.

"Matron" writes: If hospital authorities could provide sufficient accommodation to properly store patients' clothes, with previous "baking" to ensure freedom from "infection" and other equally disagreeable, if less dangerous, organisms, there is no doubt it would be advantageous to all concerned if the clothes could be kept at the hospital. I say "sufficient accommodation," for unless one can ensure the clothes being carefully put away, which entails hanging space for coats and skirts, the charge of them is not desirable. I have vivid recollections of disconsolate faces when a coat or skirt was brought to a patient full of creases, the result of being done up in a parcel for several weeks, and the angry exclamation: "You don't expect me to wear clothes like these," followed by a long tirade against those who have done their best with the limited space at hand. "E. W." seems lacking in ordinary common sense. What would have been easier than for her to have sent back the "shawl" and "bed jacket" by Parcel Post, the patient's friends would surely have made no objection to paying the 4d. or 8d. carriage? I cannot agree with "E. W." that there should be exemption from a general rule for nurses—even if they do live in the country—while the hard working woman who brings her relative to the hospital should be compelled to endure all the inconvenience of which she complains. One notes with amusement "E. W.'s" train of thought that because a rule existed from which she could get no exemption it was more than possible the patients would not be comfortable, and her evident relief that the patients were happy and well-cared for in spite of the existence of the obnoxious rule.

### CHRISTMAS PRESENTS.

"Contented" writes: To me it seems a poor business if the little remembrance that so many nurses have felt a great pleasure in giving to their matron is to be spoilt by the suggestion that some nurses have felt themselves obliged to contribute irrespective of any wish to do so on their own account. How must the poor matron feel? That view of the matter simply turns what one could only hope there would have been some pleasure in receiving into a cold, lifeless gift with no sincerity in it at all. What matron would not feel pained or even insulted by such a gift? Much better not to give it, if that is all that we can do. But is it all? Think of the number of nurses who admire and truly care for their matron. To us it is simply a delight that we are allowed that one privilege, for such we feel it. I have been a nurse for some years, and I have found that those nurses who, like myself, have someone dependent upon them, and therefore to whom contributing means further self-denial, are the ones—and the only ones—who truly realise the blessedness of giving. Let those who have any uncertainty in their own mind (either through half-heartedness or monetary difficulties) simply say so, and I am sure that they will find they are thought far more of, rather than less, for their honesty. When we can do this there will be a ring of genuine sincerity in our gift, otherwise it is hopeless.

"Another Poor Charge Nurse" writes: I cannot let the question of Christmas presents pass without a word to congratulate "Sister" on the bold way she takes up the cudgels in support of the only way we poor charge nurses have of showing our love to those "near and dear" to us. I also was trained at a large provincial infirmary for five years where the lists are similar to those mentioned by a "Charge Nurse," and I know—were she to contribute to all the lists given, as a probationer—the sum would not amount to more than 5s. For where the amount is intimated the voluntary subscription is limited to 6d. on the principal lists, matron, assistant matrons; and 2d. or 3d. or the maids and porters. This mounts up among a hundred nurses; the postmen we hardly count on our hospital staff. After all the kindness we receive during the whole year it is indeed hard if even "penniless pros." are to be debarred the pleasure, probably the only luxury they allow themselves, of giving a small sum at a season when custom has made it easy. Did "Charge Nurse" feel ruffled one Christmas because she was not allowed to contribute to a ward sister's present in whose ward she was not working?

[Could the evils of this wretched system be better illustrated than by this letter?—Editor, The Hospital.]

### GENTLEFOLK AND NURSING HOMES.

"Veritas" writes: Now that there are large bequests being bestowed, it seems to me to be a good opportunity for pointing out a great need which has not hitherto been recognised. I refer to the need of nursing homes for poor gentlefolk—annuitants and others—inmates of homes where there is not any provision made for sickness, and others even less fortunate eking out a small income in one room in a poor lodging or boarding house. They are not eligible for hospitals or infirmaries. Well as the latter are now managed, they are not suitable for sufferers of gentle birth, to whom the mere fact of having to go there would be most distressing. Many have been saved from that alternative by having been received at the Hostel of God, Clapham Common. Clergymen, organists, and governesses have found a home there and loving care in their last days. Such homes might be partly self-supporting. There must be many whose friends would gladly supplement their small means for the sake of obtaining such accommodation for them. I do not plead only for the dying, but also for those who are likely to linger for an uncertain time and require skilled nursing. It is difficult to find a home for anyone suffering from blindness or heart disease, and still more so for cancer. I know from sad experience that it is impossible to find accommodation for a cancer patient under £100 a year, and it is difficult to obtain comfort and good nursing even for that sum, especially for the last stage in a severe case.

### IRRELEVANT QUESTIONS.

"Patricia" writes: You must excuse my trespassing on your valuable space, but I should like to bring to your notice the manner in which the religious question is constantly being brought forward at so many Poor Law infirmaries lately. At the recent appointment for superintendent nurse of the Sculcoates Union Infirmary there were seven candidates, and each one was asked in her turn what religion she professed and if she intended getting married. Both these questions I consider quite unnecessary for the qualifications of a well-trained superintendent of nurses. In these enlightened days it is a great pity that there is not a little more broadmindedness shown, as very often the final decision as to which candidate is elected is based on the fact of her being of a religion which meets with the committee's approval. No right-minded woman would ever allow her religion (whatever it may be) to interfere with her duties. If a former nurse has been tactless enough to incur displeasure with regard to her religion, it is very hard on others who are of the same faith to be debarred from equal competition owing to this fact.

### A CONTINGENT TAX.

The Rev. F. A. Higley, of the Catholic Church, 636, Commercial Road, E., writes: May I point out that I did carry my amendment to the report of the Executive Committee of the Poor-law Associations of England and Wales. This Committee recommended that besides the initial registration fee, which was not to exceed one pound or one guinea, I forget which, there should also be an annual registration fee. I pointed out that medical men did not have to pay an annual fee, and that I did not think that it was fair that nurses with small salaries should be called upon to pay an annual tax. Moreover, I pointed out that every year at least 1,000 nurses would be added to the register if it came into existence, and that £1,000 per annum for registration fees ought to be enough for the maintenance of a register. My amendment was carried *nem. con.* I have seen this mistake in the Poor-law journals, but I should like to see it corrected in The Hospital.

[The report of the meeting on which we commented contained the statement which Mr. Higley now corrects.—Editor, The Hospital.]

### ANOTHER STRANGE DISMISSAL.

"Busy Bee" writes: With regard to the letter from A. E. Middleton in your issue for December 15 *re* "A Strange Dismissal," I think there are a great many dismissals of that kind, which the victims keep quiet because they are afraid that people who do not know them may think that there was some good reason for their dismissal which has not been mentioned. A nurse whom I know was about four years ago nursing in a Home where one other nurse was kept in the North of England; after she had been there three years she asked for an increase of salary, which was promptly granted, and within one month of that time she had a letter from the Secretary asking her to resign, but giving no reason. She went to see the Secretary, but all he could say was that the Committee thought that a change would be beneficial. A testimonial was given her when she left by the same Secretary to the effect that Committee, doctors, and patients were satisfied with her, the conclusion being: "I cannot speak too highly of her as a district nurse." Another, who had charge of a Home, was during the last year asked to resign because the Committee said she was not strong enough for their work, although she had done as much as her predecessor and had only been off duty one week through influenza. She had even been too busy to take the month's holiday to which she was entitled.

### BABY'S COMFORTER.

"A Lover of Babies" writes: May I say in answer to a "Trained Nurse" that I too am a trained nurse doing only maternity work, and have had six years' private experience, doing nine to ten cases every year. Therefore, I have a good deal to do with infants, especially as in these days when a nurse stays often five or six weeks after the confinement, and I think she does her little charges a great wrong to introduce the so-called "comforter" to them. She may not abuse what she calls the use of it; but what does the permanent nurse do who comes after her? The monthly nurse only uses it for a month, or a week or two longer. The permanent nurse takes it on and uses it regularly for two and even four years, and I consider the monthly nurse is to blame for this "abuse" of the comforter for introducing it into the house, and giving the mother the idea that as trained nurses approve of it, therefore it must be right. And does the permanent nurse keep it in boracic? My experience is that when a baby cries it cries for a reason, and it is the nurse's duty to find out that reason; and if a baby is well and properly fed and kept warm it does not require to suck itself to sleep, and it will not cry. I often feed a baby in its cot, sitting by it to see that it takes it properly, as is done with a sick infant that cannot be picked up, and I find that when the child has had its feed it puts the teat out and refuses to suck any more; and the same can be said of the "home-fed" infant—when it has had sufficient it drops the nipple. It is not natural for a baby to suck except for feeding. Before I took up maternity work, and after I left hospital, I used to nurse sick babies, and have often found that the digestion of the misfed infant worked perfectly well when the "comforter" was behind the fire; and my little patients, with a little real comforting, did not cry without it, and in two or three days did not even miss it. Since I read Dr. Bedley's article I have particularly noticed older children who have had comforters, and found the deformity he mentions in nearly every one of them.

### THE STANDARD OF NURSING.

"A. M." writes: Having read "Mater's" letter, I can sympathise with her daughter. I have myself for years had a wish to become a hospital nurse. A friend who, previous to her marriage, was charge nurse in a hospital, having begun at the bottom of the ladder by working as wardmaid, advised me to do the same. I have for three years worked as wardmaid, and now find that all my replying to advertisements for a probationership seems of no avail. Why not give the wardmaid a chance? There are some respectable, energetic, and intelligent girls to be found working as wardmaids, who, if they were to receive help from those who have the power, would, I am sure, give satisfaction.

### WAIHI HOSPITAL.

"One of the Staff" writes from the Hospital, Waihi, New Zealand, under date of October 9, 1906: I think it is a pity that your wholesale condemnation of the mode of procedure *re* the trustees and remaining charge nurse should have been given publicity in your issue dated September 1st. The last report was misrepresented, and was not associated with the original trouble—that of a nurse being found asleep on duty. Had you in hand the gist of the troubles, then you would have been in a position to judge rightly or wrongly. The origin of the trouble which you reported was associated with the dispositions of those immediately concerned, whose resignations have given tone and discipline to the staff of the Waihi Hospital, at whose hands it would for all time have suffered.

### Appointments.

Eton College Sanatorium.—Miss Gertrude Tomlinson has been appointed matron. She was trained at St. Bartholomew's Hospital, London, and has been sister of the Pay Wards at the Great Northern Central Hospital, London.

Great Yarmouth Hospital.—Miss Lila L. S. Dawson has been appointed matron. She was trained at University College Hospital, London. She has since been ward sister at the City Hospital, Birmingham, temporary matron at Little Bromwich Hospital, assistant lady superintendent at Cork Street Fever Hospital, Dublin, and matron of the City Hospital, East Liverpool. She has also done private, hospital, and district nursing on the staff of St. John's Home, Norfolk Street, Strand.

Isle of Wight Workhouse Infirmary.—Miss J. E. Lynn has been appointed superintendent nurse. She was trained at Birmingham Workhouse Infirmary, and has since been charge nurse at the Workhouse Infirmary, Christchurch, Hants. She holds the C.M.B. certificate.

Leith Public Health Hospital, Edinburgh.—Miss Ella B. Paterson has been appointed sister. She was trained at the City Hospital for Infectious Diseases, Edinburgh, and the Western Infirmary, Glasgow. She has since been charge nurse of the Enteric Wards in Knightswood Hospital, Glasgow.

Samaritan Hospital for Women, Liverpool.—Miss May Wylie has been appointed matron. She was trained at the Royal Southern Hospital, Liverpool, where she afterwards took sister's holiday duty. She has since been night superintendent and ward sister at the Hospital for Women, Liverpool, where she also acted as lady superintendent (locum tenens) for six months. She was subsequently night superintendent at the Rotherham Hospital and Dispensary.

Sculcoates Workhouse Infirmary, Hull.—Miss Fannie Brotherton has been appointed superintendent nurse. She was trained at Toxteth Park Poor-law Infirmary, Liverpool, and has since been superintendent nurse at Belper Poor-law Infirmary.

Sherburn Hospital, Durham.—Miss Margaret Emma Price has been appointed staff nurse. She was trained at the Leamington and South Warwickshire Hospital, and has done private nursing.

Sussex Eye Hospital.—Miss Jessie Shaw has been appointed matron. She was trained at the Hahnemann Hospital, Liverpool, and has since been sister at the Central London Ophthalmic Hospital, sister at Birmingham Eye Hospital, and sister at Bradford Eye and Ear Hospital.

Woodford Jubilee Hospital, Woodford Green, Essex.—Miss Winifred Tucker has been appointed matron. She was trained at the Evelina Hospital, Southwark, and at King's College Hospital, London. She has since been sister at the latter institution for five years.

### Holiday Amusements.

### "A MIDSUMMER NIGHT'S DREAM" AT THE ADELPHI THEATRE.

This particular play of Shakespeare's is always one of the most interesting to criticise, because it raises more than one problem : the problem whether "A Midsummer Night's Dream" should be acted at all, and if so what form the production should take.

The verse which Shakespeare employs throughout this comedy is so lyrical that it inevitably makes an appeal more to the reader than to the spectator. For in lyric verse a true appreciation is made more difficult when the measure of the verse has to be sacrificed to the accentuation and emotion of the actor. Strictly speaking, therefore, lyric verse is unsuitable for the actor unless he decides to declaim, which is a decision *not* to act. Such a decision can hardly be expected from the artist whose triumph lies not in the vocal reproduction of lyrical periods, but in the emotional rendering of verse purely from the intellectual as opposed to the strictly emotional point of view.

The actor appeals to the intellect quite as much as to the emotions of his audience ; at all costs he must make himself understood. The lyricist, on the other hand, relies for his effect not on the clearness, but on the musical quality of his words. Thus, it is evident, if it is decided to act a lyrical drama at all, exceptional self-restraint is required from the actors. In a word the actor is required not to act.

Supposing, however, such a lyrical drama is produced, how far should modern science assist the imagination by a realistic imitation of the scene? It is, of course, clear that no possible beauty which the stage-management can impart should be absent, but this is not the same as saying that a lavish production, in the common sense of the word, is essential. Those who advocate every possible embellishment to the production of "A Midsummer Night's Dream" are apt to forget that all good art contains an element of self-restraint, in fact, that in all good art something must be left to the imagination. In this way a lavish scenic display runs the enormous risk of entirely supplanting the imagination, of leaving the imagination idle, of, in fact, becoming actually a bore.

Mr. Oscar Asche in his production has not attempted the impossible : his fairies do not fly. However, like all modern stage-managers, he has seen fit to alter Shakespeare's arrangements by merging scenes and acts together to suit his requirements. If we must quarrel with him it is because he has seen fit to omit Dr. Arne's famous rendering of "Where the Bee Sucks"—is this as a protest against the frequent singing of that song during the play by an actor in a recent rendering of the piece? Again, in the last scene the play performed by Bottom, Quince, and the rest showed a tendency to develop into pure burlesque. But Mr. Asche certainly achieved his object, for the audience thoroughly enjoyed themselves.

### Christmas Presents.

### ERASMIC SOAP AND PERFUME.

Among welcome presents at Christmas may confidently be mentioned a pretty box of Erasmic Toilet Soap or a case of Erasmic Herb Perfume prepared under the auspices of the Erasmic Company, Limited, Warrington. Both preparations have the recommendation of being not only fragrant and refreshing, but free from any ingredients likely to be injurious to the skin.

### THE "PERI-LUSTA" FANCY-WORK COMPETITION.

Those who are competing in the "Peri-Lusta" grand fancy work competition will be interested to learn that all the pieces of work for which prizes have been awarded are to be publicly exhibited. Messrs. James Spence and Co., of St. Paul's Churchyard, London, have arranged to give up one of their windows to the exhibition from February 11th to the 16th, inclusive. There the successful competitors will be able to gaze proudly on their handiwork, while the unsuccessful can study effects, and purchase at once the needful materials—as Messrs. Spence themselves keep all the "Peri-Lusta" threads—to go and do likewise. The judge is Mrs. Humphry, "Madge" of *Truth*, and every lady who has competed will receive by post a list of the prize-winners.

### A Book and its Story.

### STUDENT LIFE IN EDINBURGH.*

Dr. Margaret Todd's—Graham Travers'—clever novel is one that should attract Hospital readers, for its interest depends mainly on the character of Judith Lemaistre, a girl who is studying dispensing in Edinburgh. She lives a frugal life in a flat in an unfashionable quarter, and spends her spare moments in helping any stray persons she encounters, in preference to passing time in a round of unsatisfying amusements. Amongst other characters of interest are Dr. Heriot, a physician, Dugald Dalgleish, a student at the University of Edinburgh, Thatcher, a presbyterian minister, and Ianthe Brooke, an actress

Dugald Dalgleish has come up for his first term at the 'Varsity. His father, who is dead, had been the minister of a country kirk. Dugald has won a bursary, otherwise, like many of his fellow-students, he could not have borne the expense of a university career. It is the autumn term, and Dalgleish has come to a meeting of the Young Men's Debating Society. "A meeting composed of students drawn from all the faculties of the university; . . . there were clerks, tradesmen, teachers, artisans, an accountant or two, and a number who did not lend themselves to obvious classification. . . . While the autumn wind sobbed wildly in the chimney and made dismal music among the rafters of the great chapel overhead, the Society was putting forth a rich promise of spring. There is, of course, an unfailing spring which comes to the intellectual world in October as surely as the celandine and primroses crop out in the meadows in March. Who that has lived in a university town as the autumnal days draw in has not felt the throb of new life; has not heard, as it were, the very rise of that sap? But something more than an unfailing spring had come to our society. Some ardent spirit had risen up of late and had called all its forces into activity and conflict. . . . Oh yes; of course it was pathetic and *borné* and provincial, that little assembly; so much youth and dogmatism and shyness, ambitions and dreams so far out of touch with sober probabilities. But after all it was alive, and life is the thing that counts." After the meeting, where Dugald had attracted attention by expressions of approval of the sentiments of a broad-minded speaker on the subject of religious toleration, he leaves his fellow-students and makes his way rapidly up Princes Street, imbued with the spirit of imagination; he lives for the time being in the past, and conjures up events of history. "The rain had ceased, and far up behind the castle the clouds had made a ragged window for the moon. The laughter of her light was in the boy's eyes, and the dance of the wind in his veins. This was Edinburgh indeed! . . . Brave little Edinburgh . . . how he loved to picture her as she was in the good old days! And now on this wild night the past was still alive; the air was full of ghosts; the thrill of vague rumour was in the boy's ears. The sound of far-off tumult—did it come from Kirk-o-Field? He could almost have stopped a hurried passer-by to ask whether Darnley had been murdered indeed." On returning to his rooms he goes out again to post a home letter, when, in mounting the stairs to the flat, he is surprised to see a woman ascending the stair in front of him. "His heart beat a trifle quicker. . . . She was young, to judge by her step, and even in that dim light there was something in the pose of her figure that pleased him. . . . And was she frightened—as frightened she well might be—to hear a man's step behind her on the lonely stair? She did not quicken her steps, and she held herself as one who finds the world a home. Steadily and sedately she went on to the second floor, and paused at the door opposite his. He could catch a glimpse of her face now in the moonlight if he tried. But no. She seemed to open and shut in a moment, and she was gone, and he stood on the landing alone. Who was she? What was she? The night was very late. This stair was no abode of gentlefolk, yet gentle she seemed to him."

Miss Brown, Dugald's landlady, "unquestionably pious" and, like her rooms, "fairly clean in a superficial way," figures prominently and volubly at this period, on occasions, when she has had recourse to "a wee drappie to strengthen her nerves." No story of middle-class Scotch life seems to be complete without pictures of religious meetings, and the present one has many pages devoted to them and to theological discussions that have no special interest to readers on this side of the Border, except to those in sympathy with their subject. But as an important element in the life of the class represented, they may be necessary to a correct understanding of it. The next time Dugald meets his neighbour, Judith Lemaistre, he follows her home, and finds that she has been annoyed by the attentions of a fellow-student—a stranger to her. They enter into conversation for a few minutes, and she accepts a slight courtesy that he is able to offer her. To-night he sees her face plainly. "The result was perplexing in the extreme. He told himself he was disappointed, and yet his curiosity was not dulled. She was not pretty, but her face was alive and full of purpose; she was not a mere girl, and yet a painter might have chosen her as a type of youth." Judith Lemaistre's flat was opposite that of Dugald's landlady. She was served in it by an elderly woman whom she had befriended. One evening her benevolent instincts led her across the landing to trace the source of some cries of pain. She finds Miss Brown has burnt her hand. The accident arises from her having "taken something to keep out the cold." She dresses the hand, and is leaving when she comes face to face with Dugald, looking very tired. "All the mother in Judith awoke at sight of him. A young face does not usually express fatigue, but Dalgleish looked very tired. 'Miss Brown has had a nasty little accident,' she said, 'and the good things she was getting ready for you are spoilt. I think—perhaps—you had better come and have supper with me.' She gave the invitation half reluctantly, as if in the circumstances it was really the only sensible thing to do. Was he not a mere boy, and she—oh, such an experienced woman of the world" Dugald accepts the invitation, and finds himself in a contrasting atmosphere to that of his own quarters. Judith Lemaistre's flat was spotlessly clean and rather aggressive in its simplicity, and it contained a few things to which the eye wandered back with increasing pleasure. To-night there were some yellow wallflowers in a blue jar, which harmonised very kindly with the books and pictures among which they found themselves; and the austere aspect of the room gave them an added piquancy and charm. There was something austere, too, in the aspect of Judith herself. Without making her actually pretty, Nature had finished her off very neatly. . . . She was not one of those people who drag about a body for which they seem to hold themselves only half responsible." Judith's benevolent intentions towards Dugald are interrupted by a sudden summons to Italy from her sister, whose husband has had a seizure. Here she meets Dr. Heriot. There are some fascinating chapters on Italy, and this part of the book, free from the cramped circle in which the earlier part moves, is the more attractive. The character of Ianthe Brooke, the actress, is a very sweet one. She is a sister of the minister, Thatcher, who solves the problem, of the lengths to which religious toleration and research may carry a student, by becoming a Roman priest himself.

* "Growth." By Graham Travers. Archibald Constable and Co. 6s.

### Notes and Queries.

**REGULATIONS.**

**The Editor is always willing to answer in this column, without any fee, all reasonable questions, as soon as possible.**

**But the following rules must be carefully observed.**

**1. Every communication must be accompanied by the name and address of the writer.**

**2. The question must always bear upon nursing, directly or indirectly.**

**If an answer is required by letter a fee of half-a-crown must be enclosed with the note containing the inquiry.**

*Trained Nurse.*

(140) I am anxious to start a small nursing home with a friend. Could you tell me of an inexpensive book which would advise me how to set about it, or give me a little help in Notes and Queries?—*Trained Nurse.*

There is no book published such as you ask for. We have seen so much disaster through nurses setting up a nursing home, that we earnestly advise you not to do so unless you have a large connection amongst doctors in the place where you propose to establish your home, which should possess few similar establishments, or, better still, none; and at least sufficient capital to meet all expenses during three years.

*School Nurse.*

(141) Can you tell me the best way to secure a post as nurse in a boys' school?—*Kelvinside.*

Advertise, especially in scholastic journals.

*Massage.*

(142) Can you tell me the name of a good school for massage?—*Stourport.*

You cannot do better than write for advice to the Society of Trained Masseuses, 12 Buckingham Street, Strand, W.C.

*Private Nurses' Institute.*

(143) Will you kindly give me the names and addresses of some good private nurses' institutes in West or South London?—*Anxious One.*

We do not give addresses of private institutions; but there is a list of medical homes in a little book called "Medical Homes for Private Patients," price 7d. post free, to be obtained from The Scientific Press, 28 Southampton Street, Strand, W.C., which might help you.

*Children's Nurses.*

(144) Will you tell me (1) the address of the house in Liverpool where ladies are trained as children's nurses; and (2) if there is any other house where such training is given besides Liverpool and the Norland Institute?—*Dublin.*

(1) The Liverpool Ladies' Sanitary Association, 8 Sandon Terrace, Liverpool; (2) also the Princess Christian Training College, 19 Wilmslow Road, Withington, Manchester, or the Training Home in connection with the Rochester Diocesan Association, Flora Villa, The Wrythe, Carshalton.

*A Colonial Nurse.*

(145) Can you tell me where I could be treated in London for kidney trouble?—*Inquirer.*

We feel sure if you write to the Superintendent of Guy's Hospital, S.E., or one of the other hospitals, that you will be received if you explain your case.

*Where to Train.*

(146) I am anxious to become a probationer nurse, and should like to go to Newcastle-on-Tyne in January. Is it better to start in a hospital, or does it make any difference if I start in a workhouse infirmary?—*Leeds.*

At the Royal Infirmary, Newcastle-on-Tyne, you would get a thoroughly good training, with three years' course and certificate. You can start your nursing career quite as well in a Poor-law infirmary, if it has a recognised school of training.

*Holiday Work.*

(147) Where can I obtain employment for a month during Christmas holidays—general training?—*Naval Nurse.*

Only by inquiring of friends or by advertising.

**Handbooks for Nurses.**

| | Post Free. |
|---|---|
| "How to Become a Nurse: How and Where to Train." | 2s. 4d. |
| "Nursing: its Theory and Practice." (Lewis.) ... | 3s. 6d. |
| "Nurses' Pronouncing Dictionary of Medical Terms." | 2s. 6d. |
| "Complete Handbook of Midwifery." (Watson.) ... | 6s. 4d. |
| "Preparation for Operation in Private Houses." ... | 0s. 6d. |

Of all booksellers or of The Scientific Press, Limited, 28 & 29 Southampton Street, Strand, London, W.C.

### For Reading to the Sick.

CHRISTMASTIDE.

The time draws near the birth of Christ:
The moon is hid; the night is still;
The Christmas bells from hill to hill
Answer each other in the mist.

Peace and good will, good will and peace,
Peace and good will, to all mankind.

They bring me sorrow touched with joy,
The merry, merry bells of Yule.

Rise, happy morn, rise, holy morn,
Draw forth the cheerful day from night:
O Father, touch the east, and light
The light that shone when Hope was born.

*Tennyson.*

The Christmas bells ring out once more and re-echo the angels' greeting. "Glory to God in the Highest, and on earth Peace, Good Will to men." Once more the silent stars shine down on a thousand homes where scattered members are for a brief time reunited to their loved ones, and little children listening to the bells that usher in the birthday of the child Christ, dream of the delights awaiting them in the celebration of His festival. "Glory to God" for His great Gift! Let those of us who at this season of universal rejoicing may be called apart to suffer, for His sake, pray for God's Peace. The Peace that passeth Understanding. May we be kept free in heart and mind from all restless longings that will make our burden harder to bear. For us, it may be, no personal home greeting is possible; and if so, we must lay our burden of isolation, which keeps us a prisoner to one room, at the foot of His Cross, remembering that He suffered loneliness for our sake. We long to join the happy home circle on Christmas Day, but sad as our case may be, there are other lonely sufferers who have no loved ones on earth left to care for and pray for them—no home where their presence is missed and longed for. For them there is special comfort in the thought of the angels and their song in the night. They will be cheered and strengthened as they dwell on the thought of those ministering spirits, those angels of consolation, whether on earth or in Heaven, who do the bidding of their dear Lord and Master, to succour and defend all in sorrow or suffering. If unable to attend the Divine observance of Christmas Day, from our little corner in the ranks of the great army of silent sufferers, we may yet give thanks to God, and keep the Festival in our hearts. If joy, as the world counts it, is denied to us, we may pray for Christmas Peace to fall on others who are lonely, or suffering, and echo the words of Adelaide Proctor's sweet poem as we realise that "Joy is like the restless day," but "Peace divine like quiet night." May God grant us this peace!—*Anon.*

It came upon the midnight clear,
That glorious song of old,
From angels bending near the earth,
To touch their harps of gold:
"Peace to the earth, good will to men,"
From heaven's all-gracious King;
The world in solemn stillness lay
To hear the angels sing.

*G. Hamilton Sears*

